# Posterior Urethral Valves: Renal Failure and Prenatal Treatment

**DOI:** 10.1155/2012/351067

**Published:** 2011-08-09

**Authors:** Daniel P. Casella, Jeffrey J. Tomaszewski, Michael C. Ost

**Affiliations:** Department of Urology, University of Pittsburgh School of Medicine, Pittsburgh, PA 15213, USA

## Abstract

Posterior urethral valves occur in 1 : 5000 live births. Despite the high prevalence, the few children that survive do poorly, with over 50% progressing to ESRD in 10 years. The gold standard for post-natal diagnosis is voiding cystourethrography, while pre-natal diagnosis is dependent on routine screening ultrasonography. Despite the ability to identify features of bladder outlet obstruction early in fetal development, there is no consensus on how to incorporate early detection into current screening protocols. There has yet to be a marker that allows prediction of obstruction in the absence of or prior to radiographic evidence of obstruction. With our current screening strategy, the majority of interventions are performed well after irreversible damage has occurred. Improved mortality and long term morbidity from posterior urethral valves and congenital bladder outlet obstruction will likely remain unchanged until it is possible to intervene prior to the onset of irreversible renal damage. New biologic markers and improved instrumentation will allow for more effective diagnosis and intervention at earlier stages of fetal development.

## 1. Introduction

Posterior urethral valves occur in 1 : 5000 [1] live births and in approximately every 1/1250 fetal ultrasound screenings [2]. Despite the high prevalence, the most common treatment for valves identified in utero is fetal termination [3]. The few children that survive do poorly, with over 50% progressing to ESRD in 10 years [4]. Procedures for fetal bladder decompression were first introduced over 25 years ago; however they still remain technically difficult with limited proven benefit [5]. Routine prenatal ultrasound is currently not recommended until 20 weeks of gestation [6]. New understanding of the pathogenesis of renal dysplasia and long-term renal dysfunction demonstrate that changes in renal development and architecture may begin to occur as early as 14 weeks of gestation (7,13,40). In animal models of complete outlet obstruction, renal damage is significant and irreversible in as little as 30 days [7]. With our current screening strategy, the majority of interventions are performed well after irreversible damage has occurred. Improved mortalility and long-term morbidity from posterior urethral valves and congenital bladder outlet obstruction will likely remain unchanged until it is possible to intervene prior to the onset of irreversible renal damage.

## 2. Embyologic Development

The definitive kidney of a human embryo, the metanephros, begins development on the 28th day of gestation when the ureteral bud comes in contact with the metanephric mesenchyme. Through reciprocal mesenchymal/epithelial interactions, the collecting system is formed from the branching urteric bud and the nephron from the overlying mesenchyme. Fetal urine production begins as early as 8 weeks, and by 12 weeks, the collecting system including ureters and urinary bladder has formed. It is not until the 13th week that smooth muscle and autonomic innervation of the bladder are complete [8]. While further maturation of existing nephrons occurs after birth, nephrogenesis is completed by 34 weeks of gestation [9].

## 3. Pathogenesis of Renal Dysfunction

There is no single genetic mutation or biologic model that reproduces the phenotype of posterior urethral valves or congenital bladder outlet obstruction. In early work with fetal sheep, surgical obstruction caused hydronephrosis within one week and resulted in dysplastic changes at term [10]. Further studies confirmed the presence of dysplastic changes and altered nephrogenesis in kidney's exposed to outlet obstruction during development [11–13]. Altered cellular differentiation, phenotypic changes in glomerular cells [14], apopotosis, and increased oxidative stress [15] contribute to decreased nephrogenesis and renal dysplasia. A key early event appears to be distention and mechanical stretch of the dilated collecting system, leading to activation of the apoptotic and inflammatory cascades [16]. 

The decreased number of total nephrons present at birth leads to hyperfiltration injury, exacerbation of the underlying inflammatory process, renal fibrosis, and ultimately renal failure [17, 18]. The early impact of altered differentiation and activation of the apoptotic cascade clearly illustrates that the molecular mechanisms and alterations in renal architecture which underlie progressive renal failure are established in utero.

## 4. Pathophysiology of Posterior Urethral Valves

Posterior urethral valve (PUV) is the most common cause of obstructive uropathy leading to renal failure in male neonates [19]. Although the true incidence of PUVs is not precisely known, PUV is estimated to occur in 1 : 5000 live births [1]. The normal male urethra is anatomically divided into the prostatic and membranous portions (posterior urethra) and the spongy or anterior urethra. The urethral crest is a mucosal ridge that gives a specific form to the posterior urethra, and on either side of the ridge is the prostatic sinus. The urethral crest continues below the verumontanum and coalesces in a small midline bridge. This membrane, extending laterally and downward, eventually vanishes [20]. 

The classic form of PUV is found in the prostatic urethra, below or proximal to the verumontanum. Although the precise embryologic mechanism of PUV remains unknown [21], four theories have been proposed to explain their development and include hypertrophy of the urethral mucosal folds, persistence and continuation of the urogenital membrane [22], abnormal development of the Wolffian or Mullerian duct [23], and fusion of the verumontanum or the posterior urethral roof epithelium [24].

## 5. Diagnosis

### 5.1. Radiographic Diagnosis

The gold standard for post-natal diagnosis is voiding cystourethrography, while pre-natal diagnosis is dependent on routine screening ultrasonography. According to ACOG guidelines, the optimal time to perform an ultrasound examination, in the absence of a specific indication for 1st trimester screening, is between 18 and 20 weeks [6]. Studies from the early 1990s revealed that the classic findings of bilateral hydronephrosis, distended bladder, dilated posterior urethra, and thickened bladder wall are missed in the majority of cases if screening ultrasound is performed prior to 24 weeks [25–28]. With the significant improvement in ultrasound over the past decade, it is our hope that the ability to detect PUV prior to 24 weeks has improved; however there are no contemporary series addressing this question. Between 18 and 22 weeks, 2–5% of fetuses will be found to have dilation of the renal pelvis. Hydronephrosis detected during the 2nd trimester resolves by term in over 80% of cases, while 40% of infants requiring surgery for urinary obstruction have no evidence of hydronephrosis on prenatal ultrasound. Megacystis, a potential early marker of bladder outlet obstruction, can now be identified as early as 11–14 weeks of gestation [29]. 75% of fetuses with megacystis (from 7–15 mm) will have a normal karyotype and 90% will spontaneously resolve, while the remaining 10% will progress to obstructive uropathy. There have also been reports of megacystis diagnosed at 11 weeks progressing to the development of new bilateral hydronephrosis by 13 weeks [30, 31]. Megacystis may be an early finding in outlet obstruction and the hydronephrosis associated with outlet obstruction may be identifiable earlier than previously thought. Despite the ability to identify features of bladder outlet obstruction early in fetal development, there is no consensus on how to incorporate early detection into current screening protocols.

### 5.2. Biochemical Diagnosis

Fetal urinary electrolytes are the only biological markers that have been studied as an in utero indicator of renal function and predictor of long-term function. Despite extensive reporting in the literature and widespread utilization of fetal urinary electrolytes, a recent meta-analysis demonstrated no significant predictive value of urinary electrolytes in the diagnosis of obstructive uropathy or long-term renal preservation [32]. Studies aimed at classifying hydronephrosis have also attempted to identify biological markers of functionally significant obstruction. Given the new and more extensive understanding of inflammation's role in the etiology of renal damage, it is not surprising that TGF-*β*1, TNF-alpha, and MCP-1 have all been found to be elevated in children with UPJ obstruction [33, 34]. Proteomic analysis of children with UPJ obstruction identified several collagen degradation products predictive of renal obstruction severity [35]. To date, none of these markers have been studied in the fetal environment or prior to the radiologic diagnosis of obstruction. There has yet to be a marker that allows prediction of obstruction in the absence of or prior to radiographic evidence of obstruction.

## 6. Treatment

The most common therapy for antenatal bladder outlet obstruction remains pregnancy termination [3]. Early therapies to relieve fetal urinary obstruction were performed through open fetal ureterostomies and cutaneous vesicostomies [36]. Given the risks of open fetal surgery, a minimally invasive percutaneous approach was adopted. In what has now become the most common approach, a trocar is placed into the fetal bladder through which a double-j catheter is positioned allowing drainage of fetal urine into the amniotic fluid cavity. Percutaneous stent placement was widely used initially; however reviews 5 years after its implementation reported a 4.6% fetal mortalilty rate and a complication rate of 44% [37]. Reports of high complication rates coupled with poor fetal long-term survival led to decreased utilization of percutaneous drainage, and its use remains experimental. In a more contemporary series, when placed between 20 and 28 weeks of gestation, shunts have limited obstetric complications; however there are still a high percentage of mechanical complications [38]. 

Fetal cystoscopy through the same needle trocar used to place vesicoamniotic shunts was first reported in 1995 [39]. With advances in endoscopic instrumentation, there have now been case reports of fetal cystoscopy and valve ablation [40]. 

Despite being introduced over 25 years ago, literature regarding antenatal intervention consists of case reports and small series. In a recent meta-analysis Morris et al. [5] identified 20 studies of sufficient quality, representing a total population of 369 fetuses, of which 261 had undergone antenatal intervention. The majority underwent vesicoamniotic shunting; however patients undergoing cystoscopic ablation or open procedures were also included. The authors report an overall survival advantage when intervention is compared to no intervention. However, when renal function was examined, the odds of being born with normal renal function were decreased in the population undergoing fetal intervention. It has been postulated that this finding is due to the survival of fetuses that would otherwise have died without antenatal intervention. While intriguing, this study includes no randomized controlled trials (RCTs) and key data such as the age at which intervention was performed and the indications for antenatal intervention were largely unavailable. 

The percutaneous shunting in lower urinary tract obstruction (PLUTO) trial [41] is an international multicenter study which began enrolling patients in 2008. The goal is to identify 150 male fetuses with ultrasound evidence of lower urinary tract obstruction and to randomize them to undergo vesicoamniotic shunting or observation. While the first RCT will be a valuable study with appropriate endpoints, several key variables such as standardized criteria for ultrasound diagnosis and fetal age were not included in the study design and may limit meaningful conclusions if these variables are not controlled for properly.

## 7. Conclusion

Congenital outlet obstruction continues to be a significant source of fetal morbidity with little progress in its prenatal treatment or management over the last 25 years. Advances in the understanding of pathogenesis make it clear that the mechanical stretch of a dilated system leads to decreased nephrogenesis and renal dysplasia through altered differentiation, apoptosis, and inflammation. In fetal lamb models bladder outlet obstruction leads to acute tubular necrosis within 48 hours and dilation of developing tubules and interstitial widening within 5–7 days of obstruction [42]. When bladder outlet obstruction, in fetal lambs, induced on day 60 is relieved with a vesicoamniotic shunt on day 90, nephrogenesis is recoverable. Kidneys in which the obstruction was reversed will have a 5% loss of total nephrons compared to 20% loss in those with persistent obstruction [7]. 

Fetal urine production begins as early as eight weeks; however a muscular bladder capable of transmitting increased intravesicle pressures to the developing kidney is not developed until 13 weeks. Based on animal models and the new understanding of the effect of obstruction on nephrogenesis, it is likely that renal damage in congenital bladder outlet obstruction occurs as early as 14 weeks. The crucial period for lung development is also between 16 and 25 weeks. 14 days of persistent severe oligohydraminos prior to 25 weeks is associated with a fetal mortality of 90%. 

 It is now possible to identify radiographic changes indicative of outlet obstruction as early as 11–14 weeks; however our current screening recommendations are initiated four to six weeks after this key developmental stage. New biologic markers and improved instrumentation will allow for more effective diagnosis and intervention at earlier stages of fetal development. Until intervention close to the onset of obstruction is possible, there will likely be little impact in the overall morbidity and progressive loss of renal function in infants with congenital bladder outlet obstruction.
